# Optimization of salicylic acid concentrations for increasing antioxidant enzymes and bioactive compounds of *Agastache rugosa* in a plant factory

**DOI:** 10.1371/journal.pone.0306340

**Published:** 2024-07-25

**Authors:** Vu Phong Lam, Dao Nhan Loi, Juhyung Shin, Lee Kyeong Mi, Jongseok Park

**Affiliations:** 1 Department of Horticultural Science, Chungnam National University, Daejeon, South Korea; 2 Department of Agronomy, Tay Bac University, Son La, Vietnam; 3 Department of Bio-AI Convergence, Chungnam National University, Daejeon, South Korea; University of Brescia: Universita degli Studi di Brescia, ITALY

## Abstract

Salicylic acid (SA) plays a crucial role as a hormone in plants and belongs to the group of phenolic compounds. Our objective was to determine the optimal concentration of SA for enhancing the production of bioactive compounds in *Agastache rugosa* plants while maintaining optimal plant growth. The plants underwent SA soaking treatments at different concentrations (i.e., 0, 100, 200, 400, 800, and 1600 μmol mol^−1^) for 10 min at 7 days after they were transplanted. We observed that elevated levels of SA at 800 and 1600 μmol mol^−1^ induced oxidative stress, leading to a significant reduction across many plant growth variables, including leaf length, width, number, area, shoot fresh weight (FW), stem FW and length, and whole plant dry weights (DW) compared with that in the control plants. Additionally, the treatment with 1600 μmol mol^−1^ SA resulted in the lowest values of flower branch number, FW and DW of flowers, and DW of leaf, stem, and root. Conversely, applying 400 μmol mol^−1^ SA resulted in the greatest increase of chlorophyll (Chl) a and b, total Chl, total flavonoid, total carotenoid, and SPAD values. The photosynthetic rate and stomatal conductance decreased with increased SA concentrations (i.e., 800 and 1600 μmol mol^−1^). Furthermore, the higher SA treatments (i.e., 400, 800, and 1600 μmol mol^−1^) enhanced the phenolic contents, and almost all SA treatments increased the antioxidant capacity. The rosmarinic acid content peaked under 200 μmol mol^−1^ SA treatment. However, under 400 μmol mol^−1^ SA, tilianin and acacetin contents reached their highest levels. These findings demonstrate that immersing the roots in 200 and 400  μmol mol^−1^ SA enhances the production of bioactive compounds in hydroponically cultivated *A*. *rugosa* without compromising plant growth. Overall, these findings provide valuable insights into the impact of SA on *A*. *rugosa* and its potential implications for medicinal plant cultivation and phytochemical production.

## Introduction

*Agastache rugosa*, commonly referred to as Korean mint, is a perennial herb classified within the Lamiaceae family, and it is extensively cultivated in China, Korea, Vietnam, Japan, and Thailand primarily because of its aromatic properties and potential medicinal and therapeutic benefits. In addition, Korean mint is highly valued as a functional food because of its distinct flavor and potential health-promoting properties [1]. *A*. *rugosa* possesses notable quantities of pharmacologically significant bioactive compounds, including tilianin, rosmarinic acid (RA), and acacetin, which contribute to its valuable medicinal properties [2, 3]. RA exhibits diverse therapeutic properties, functioning as an antimicrobial, anti-allergic, anti-diabetic, anti-inflammatory, immunomodulatory, and hepato-and renal-protective agent [4]. In contrast, tilianin is known for its cardioprotective [5], whereas acacetin exhibits a broad spectrum of pharmacological activities [6]. Among the various parts of *A*. *rugosa*, the flowers contain the highest concentrations of RA, tilianin, and acacetin [2]. The bioactive compounds found in *A*. *rugosa* make it a promising candidate for developing effective medicines. The cultivation of *A*. *rugosa* in southern Korea primarily occurs in wild areas or open fields, where the instability of environmental factors can lead to variable harvest yields and unstable bioactive compound levels. Due to its cultivation in relatively small areas, there is a scarcity of accurate statistical data available [7]. In response to these challenges, efforts have been made to cultivate *A*. *rugosa* in closed-plant factory systems to achieve consistent plant quality and to formulate standardized growth conditions [8, 9]. Plant factories use multi-layer shelves to provide larger cultivation areas. By employing precise control over temperature, lighting, CO_2_ levels, and humidity, these facilities enable year-round mass production of plants while simultaneously enhancing productivity and ensuring consistent quality [10]. Nowadays, plant factories have garnered attention as efficient systems for the production and cultivation of vegetables [10], medicinal plants [11], and herbs [12], due to their well-documented advantages.

Salicylic acid (SA) is a naturally occurring plant hormone that plays a vital role in regulating various physiological processes such as growth, development, and defense mechanisms [13]. SA plays a role in triggering plant defense mechanisms against both biotic and abiotic stresses, ultimately resulting in the buildup of secondary metabolites, including bioactive compounds [14]. The positive effects of SA in enhancing the biosynthesis of bioactive compounds in various plant species, such as *Orostachys cartilaginous* A. Bor [15], *Thevetia peruviana* [16], and *Givotia moluccana* [17]. The results of the study revealed that treating *Orostachys cartilaginous* with 100 μM SA for a duration of 48 h significantly increased the maximum production of flavonoids, phenolics, and polysaccharides [15]. The application of SA at 300 μM in *Thevetia peruviana* resulted in an increased content of flavonoid and phenolic compounds. This suggests that SA acts as an elicitor by inducing the phenylpropanoid metabolic pathway and promoting the synthesis of bioactive compounds [16]. SA significantly increases total flavonoid and phenolic contents in *Givotia moluccana* [17]. Overall, SA promotes the synthesis of secondary metabolites in plants. These compounds include various bioactive substances such as phenolic compounds, flavonoids, alkaloids, and terpenoids [18]. SA enhances the levels of phenolic compounds, antioxidant, and antihyperglycemic activities in Lamiaceae family [19]. Therefore, the application of SA enhances plant resilience against abiotic factors and enhances the nutritional value of crops.

To the best of our knowledge, no previous studies have investigated the effect of SA on the growth and accumulation of bioactive compounds in *A*. *rugosa*. Therefore, this study aimed to determine the ideal SA concentration that maximizes plant growth, bioactive compound contents, antioxidant activities, and photosynthesis in *A*. *rugosa* plants.

## Materials and methods

### Seedling growth conditions

Seeds of *A*. *rugosa*, sourced from Danong Co. Ltd. (Seoul, Korea), were planted in a tray and nurtured within a controlled environment. This environment was regulated to maintain a temperature of 21.8/18°C, a relative humidity of 70 ± 5%, and a photosynthetic photon flux density of 220 ± 10 μmol m^−2^ s^−1^, facilitated by LED lights (TL5 14W/865 Philips, Amsterdam, Netherlands), following a 16-hour light and 8-hour dark period. After two weeks of sowing, the seedlings received Hoagland solution, characterized by an electrical conductivity (EC) of 1.2 dS m^−1^ and a pH level of 6.0.

### Treatments

Twenty-eight days after sowing, the seedlings were transferred to a deep flow system within a plant factory. The environment conditions remained consistent with those employed for seedling growth. The plants were cultivated for 39 days in Hoagland solution, with a pH level maintained at around 6.5 and an EC of 2.0 dS^.^m^−1^. Seven days after transplantation, the plants were subjected to SA soaking treatments at concentrations of 0, 100, 200, 400, 800, and 1600 μmol mol^−1^ for 10 min. Thirty-nine days after transplantation, plant samples were collected for subsequent analyses.

### Growth variables

Measurements were taken for leaf length, number of flower branches, leaf width, leaf area, stem length, as well as the fresh weights of flowers, leaves, stems, shoots, roots, and root length. Furthermore, the dry weights (DW) of leaves, flowers, stems, shoots, whole plants, and roots were determined following a seven-day period of oven drying at 70°C. Fresh and dry weights were determined utilizing an electronic scale (EW220-3NM, Kern & Sohn GmbH., Balingen, Germany). The leaf area was assessed utilizing a leaf area meter (LI-3000A, Li-Cor, Lincoln, NE, USA). The flower or leaf DW ratio was calculated by dividing the flower DW, leaf area, or leaf DW by the total DW of the plants. The specific leaf area was computed by dividing the leaf area of the plant by its leaf DW.

### Photosynthetic characteristics

The SPAD value, which indicates the relative Chl content, was measured using a portable Chl meter (502; Minolta Camera Co., Ltd., Osaka, Japan). A portable photosynthetic system (LICOR 6400, Licor. Inc., Nebraska, NE, USA) was used to measure photosynthetic characteristics including net photosynthetic rate (A; μmol CO_2_ m^−2^ s^−1^), stomatal conductance (g_s_; mol H_2_O m^−2^ s^−1^), intercellular CO_2_ concentration (C_i_; μmol CO_2_ mol^−1^), and transpiration rate (E; mmol H_2_O m^−2^ s^−1^). The chamber variables were configured as follows: PPFD of 1000 μmol m^−2^ s^−1^, leaf temperature of 25°C, relative humidity of 60%, air flow rate of 500 cm^3^ s^−1^, and ambient CO₂ concentration of 400 μmol mol^−1^. Leaf gas exchange variables were determined by measuring the photosynthetic characteristics of the third fully unfolded leaf from the top.

### Photosynthetic pigments and 2,2-diphenyl-1-picrylhydrazyl (DPPH) radical scavenging activity

After harvesting, the leaves, flowers, and stems of each plant replicate were immediately submerged in liquid nitrogen and then placed in a deep freezer at -70°C. Subsequently, they were transferred to a dry freezer at -50°C using a TFD5503 freezer from IL Shinbiobase Co. Ltd, Gyeonggi-do, Korea within four days. A pestle and porcelain mortar were utilized to grind each sample, and the resulting dry powder was passed through mesh sieves for filtration. The concentrations of Chl a, Chl b, DPPH radical scavenging activity, and carotenoids (Car) were measured using an Epoch microplate spectrophotometer (EPOCH-SN; Agilent Technologies, Inc., Santa Clara, CA 95051, USA). To extract the powdered shoot samples (20 mg DW), 2 mL of 90% MeOH was added, and the mixture was centrifuged at 1308 × g for 20 min. The concentrations of Chl a, Car, and Chl b were detected at wavelengths of 652.4 nm, 470 nm, and 665.2 nm, respectively, as per the protocol described by Lichtenthaler (1987) and Lam et al. 2023 [20, 21]. The DPPH activity was conducted at a wavelength of 517 nm, following the procedure described by More and Makola, 2020 [22]. The following variables were calculated: Chl a, Chl b, Car, total Chl a + b, DPPH activity, and Chl a/b ratio.

Chl a (mg g^−1^) = (16.82 × A_665.2_ − 9.28 × A_652.4_)/10

Chl b (mg g^−1^) = (36.92 × A_652.4_ − 16.54 × A_665.2_)/10

Car (mg g^−1^) = ([1000 × A_470_−1.91 × Chl a– 95.15 Chl b]/225)/10

DPPH (%) = (A_blank_ − A_sample_)/A_blank_ ×100

Chl a: Chl b ratio (Chl a/b) = Chl a/Chl b

Total Chl a + b (mg g^−1^) = Chl a + Chl b

Where A is the absorbance at the wavelength

### Total flavonoid, total phenolic, and antioxidant enzymes

To analyze the total flavonoid contents using colorimetric techniques, we followed the method outlined by Lin and Tang (2007) and Lam et al. 2023 [21, 23]; primarily, this method involved the use of aluminum chloride (AlCl_3_). In summary, the process entailed mixing 20 mg of dry powder samples with 2 mL of 90% methanol, then subjecting them to sonication at 20°C for 20 minutes. Following the mixing process, the solution was subjected to centrifugation at 1308 × g for 20 min, keeping a temperature of 4°C. Subsequently, a 100 μL portion of the sample was combined with 300 μL of 95% (w/v) ethanol, followed by the addition of 20 μL of 10% (w/v) AlCl_3_, 20 μL of 1 M (w/v) potassium acetate (CH_3_COOK), and distilled water with 600 μL. Following a 40-minute incubation period at 22°C, the absorbance of the reaction mixture was recorded at 415 nm utilizing an Epoch microplate spectrophotometer. Quercetin was utilized as the reference compound, and a calibration curve was constructed by preparing quercetin solutions with concentrations ranging from 0 to 500 μg/mL in methanol, including concentrations of 50, 75, 125, 250, and 500 μg/mL. The results were reported as milligrams of quercetin equivalents (QE) per gram DW of the extracted powder (mg QE/g DW).

The total phenolic content of individual extracts was determined using the Folin–Ciocalteu method according to Aryal et al., 2019 [24]. To conduct the assay, the aforementioned aliquot of 100 μL sample was combined with 100 μL of a 10% Folin-Ciocalteu reagent and 1500 μL of distilled water. The resulting mixture was incubated for 5 min. Then, 300 μL of 7.5% sodium carbonate (Na_2_CO_3_) solution was added, initiating a 40-minute reaction at 22°C. Gallic acid (GA) was selected as the standard, and a 6-point standard curve ranging from 0 to 150 mg/L (0, 25, 50, 75, 100, 125, and 150 g/mL) was prepared. The samples underwent analysis utilizing an Epoch microplate spectrophotometer, with measurements taken at a wavelength of 765 nm. The results are reported as milligrams of GA equivalents per gram (mg GAE/g) of dry extract.

Subsequently, a revised protocol based on the nitro blue tetrazolium (NBT) method outlined by Hasan et al. (2022) and Kiani et al. (2021) was employed in this study [25, 26] to measure the superoxide dismutase (SOD, EC 1.15.1.1), catalase (CAT, EC 1.11.1.6), and peroxidase (POD, EC 1.11.1.7). In summary, 20 mg of dry sample was combined with 2 mL of phosphate-buffered saline (PBS) solution containing 50 mM at pH 7.0 and subjected to three rounds of sonication, each lasting 10 minutes, in liquid nitrogen. The mixture underwent centrifugation at 4°C with 1308 × g for 20 minutes. Following this, 1 mL of the extracted solution was passed through a 0.45-μm filter and then underwent analysis for SOD, POD, and CAT enzyme activities.

#### SOD enzyme

To assess the SOD activity, a reaction mixture was prepared by combining 20 μL of the sample with 52 μL of methionine, 24.5 μL of NBT, 2 μL of EDTA, 8 μL of riboflavin, and 93.5 μL of PBS at pH 7.0 and then added to a 96-well plate. Subsequently, the plate was illuminated with LED light at an intensity of 200 μmol m^−2^ s^−1^ for a duration of 8 minutes. Absorbance readings were taken at 560 nm using an Epoch microplate spectrophotometer. One unit of SOD enzyme activity was defined as "the amount of enzyme that can inhibit the photoreduction of NBT by 50% under experimental conditions. SOD activity was quantified in U mg^−1^ DW and determined using the following formula:

$$SOD\;(\backslash \%\;inhibition)=\frac{(A560\;control-A560\;sample)\;\times 100}{A560\;control}$$

$$SOD\;(Unit\;{mL}^{-1})=\frac{\%\;inhibition\;\times total\;volume}{50\;\times enzyme\;volume}$$

$$SOD\;(Unit\;{mg}^{-1}\;DW)=\frac{Unit\;{mL}^{-1}}{enzyme\;(mg\;{mL}^{-1})}$$

#### POD enzyme

A solution containing 66.6 μL of PBS with a pH of 6.1, 33.3 μL of hydrogen peroxide (H_2_O_2_), and 80 μL of guaiacol was used for the assay of the enzyme. At 25°C, the reaction was initiated by adding 20 μL of the sample extract. Enzyme activity was defined as the increase in absorbance of 1 unit of enzyme at 470 nm per min at a temperature of 25°C using an Epoch microplate spectrophotometer. The specific activity of POD was reported in U mg^−1^ DW min^−1^.


$$POD\;(\mu mol\;min¯1\;{mL}^{-1})=\frac{(A470/min)\;\times total\;volume)\;\times 1000}{26.6\;\times \;enzyme\;volume}$$

$$POD\;(\mu mol\;min^{–1}\;{mg}^{-1}\;DW)=\frac{Unit\;{mL}^{-1}}{enzyme\;(mg\;{mL}^{-1})}$$

The wavelength for absorption reading was 470 nm for 1 min and the extinction coefficient was 26.6 mM^−1^cm^−1^.

#### CAT activity

CAT activity was assessed following the procedure outlined by Aebi, 1984 [27], as depicted in the equations below. To conduct the enzyme assay, a solution comprising 193.6 μL of PBS with a pH of 7.0 and 3.4 μL of 3% H_2_O_2_ was prepared. The enzymatic reaction was initiated by adding 3 μL of the sample extract. A reference mixture did not contain any enzyme extract, referred to as the ’blank’ was placed in a spectrophotometer for 4 to 5 minutes to attain temperature equilibrium. The absorbance was measured at a wavelength of 240 nm in an Epoch microplate spectrophotometer for 3 minutes. CAT activity was quantified by determining the quantity of enzyme that decomposed 1 μM of H_2_O_2_ and expressed as μmol per milligram of DW per minute (μmol mg^−1^ DW min^−1^).


$$CAT\;(\mu mol\;{ml}^{-1}\;{min}^{-1})=\frac{(A240/min)\;\times total\;volume\;\times 1000}{43.6\;\times enzyme\;volume}$$

$$CAT\;(\mu mol\;{mg}^{-1}\;DW\;{min}^{-1})=\frac{\mu mol\;{min}^{-1}\;{ml}^{-1}}{enzyme\;(mg\;{ml}^{-1})}$$

The wavelength for absorption reading was 240 nm for 1 min, and the extinction coefficient was 43.6 M^−1^cm^−1^.

### Acacetin, tilianin, and rosmarinic acid contents and concentrations

To determine the concentrations of RA, tilianin, acacetin, and the three acacetin glycosides, acacetin 7-O-(2"-O-acetyl)β-D-glucopyranoside (acacetin 1), acacetin 7-O-(6"-O-malonyl)β-D-glucopyranoside (acacetin 2), and acacetin 7-O-(2"-O-acetyl-6"-malonyl)β-D-glucopyranoside (acacetin 3), following the procedure described by Lam et al. 2023 [21] 10 mL of 100% methanol was used to dissolve 200 mg of dry powder from flowers, leaves, roots, and stems. Before analysis, the mixture underwent 30 minutes of sonication. Following this, the blended extract was subjected to centrifugation at 1358 × g for a duration of 20 minutes. Subsequently, 1 mL of the extract solution was filtered through a 0.45-μm filter and subjected to analysis using high-performance liquid chromatography (HPLC) with a 1260 Infinity system from Agilent Technologies Inc. The mobile phase consisted of acetonitrile (solvent B) and a solution of 0.1% formic acid in water (solvent A). The gradient program was as follows: 0 to 5 minutes—20% B, 5 to 10 minutes—50% B, 10 to 20 minutes—50% B, and 20 to 22 minutes—100% B. For the analysis, an injection volume of 10 μL and a flow rate of 0.8 mL per minute were employed [28]. HPLC chromatograms were recorded at a wavelength of 330 nm. Calibration curves were constructed using standards obtained from Sigma-Aldrich (St. Louis, MO, USA).

RA, tilianin, and acacetin retention times were 11.655, 12.542, and 19.659 min, respectively. Acacetins 1, 2, and 3 were detected at 13.131, 14.485, and 15.351 min, respectively. The concentrations of these bioactive compounds, expressed as mg·g^−1^ DW, were measured in the flowers, leaves, roots, and stems of plants. The total concentration of each compound in the whole plant (mg·g^−1^ DW) was calculated using Eq (1).


$$BCx\;in\;the\;whole\;plant\;(mg∙g^{-1}\;DW)\;=\Sigma (amount\;of\;BCx\;in\;each\;part\;\times \;\%\;DW\;of\;each\;plant\;organ\;per\;unit\;DW\;of\;the\;entire\;plant).$$ (1)

BC: Bioactive compound

x: Tilianin, acacetin 1, acacetin 2, acacetin 3, or RA

The BC content of the entire plant (in mg per plant DW) was calculated by multiplying the BC concentration (in mg per g DW) by the total DW of the entire plant (g). Similarly, the BC content of each individual plant organ (in mg per organ DW) was determined by multiplying the BC concentration within that organ (in mg per gram of organ DW) by the DW of that specific plant organ (g).

### Statistical analysis

Growth variables and SPAD values were measured in six plants (n = 6) per replicate. In contrast, photosynthetic characteristics, photosynthetic pigments, total flavonoid, total phenolic, antioxidant enzymes, and BC were determined in three plants (n = 3). The statistical analysis was conducted utilizing SPSS version 20.0 software (SPSS 20, IBM Corp., Armonk, N.Y., USA). The data underwent an analysis of variance to compare the means of the qualitative factors, followed by Tukey’s test (*p* < 0.05) for comparison. Regression analysis was employed to assess the means of the quantitative factors.

## Results

### Plant growth variables

**Fig 1:**
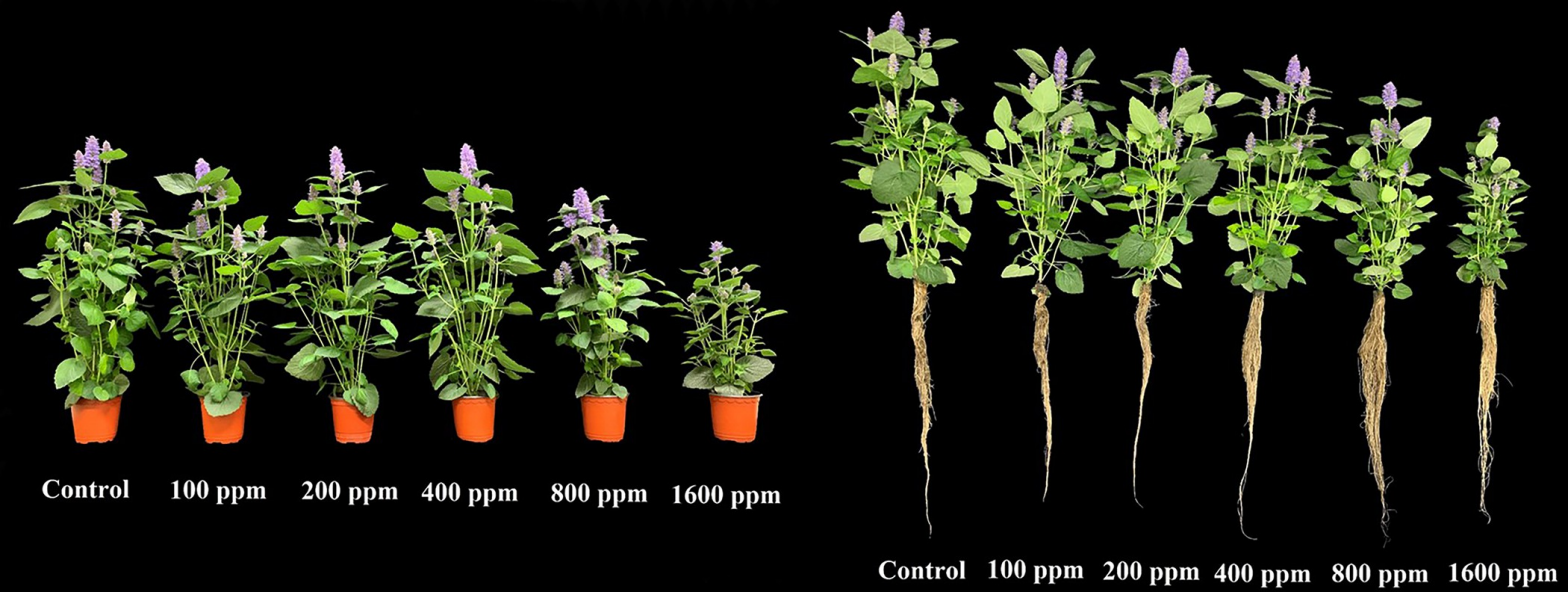
Images of *A*. *rugosa* plants growth at different salicylic acid soaking treatments (0, 100, 200, 400, 800, and 1600 μmol·mol^−1^ for 10 minutes) at 39 days after transplanting.

**Fig 2:**
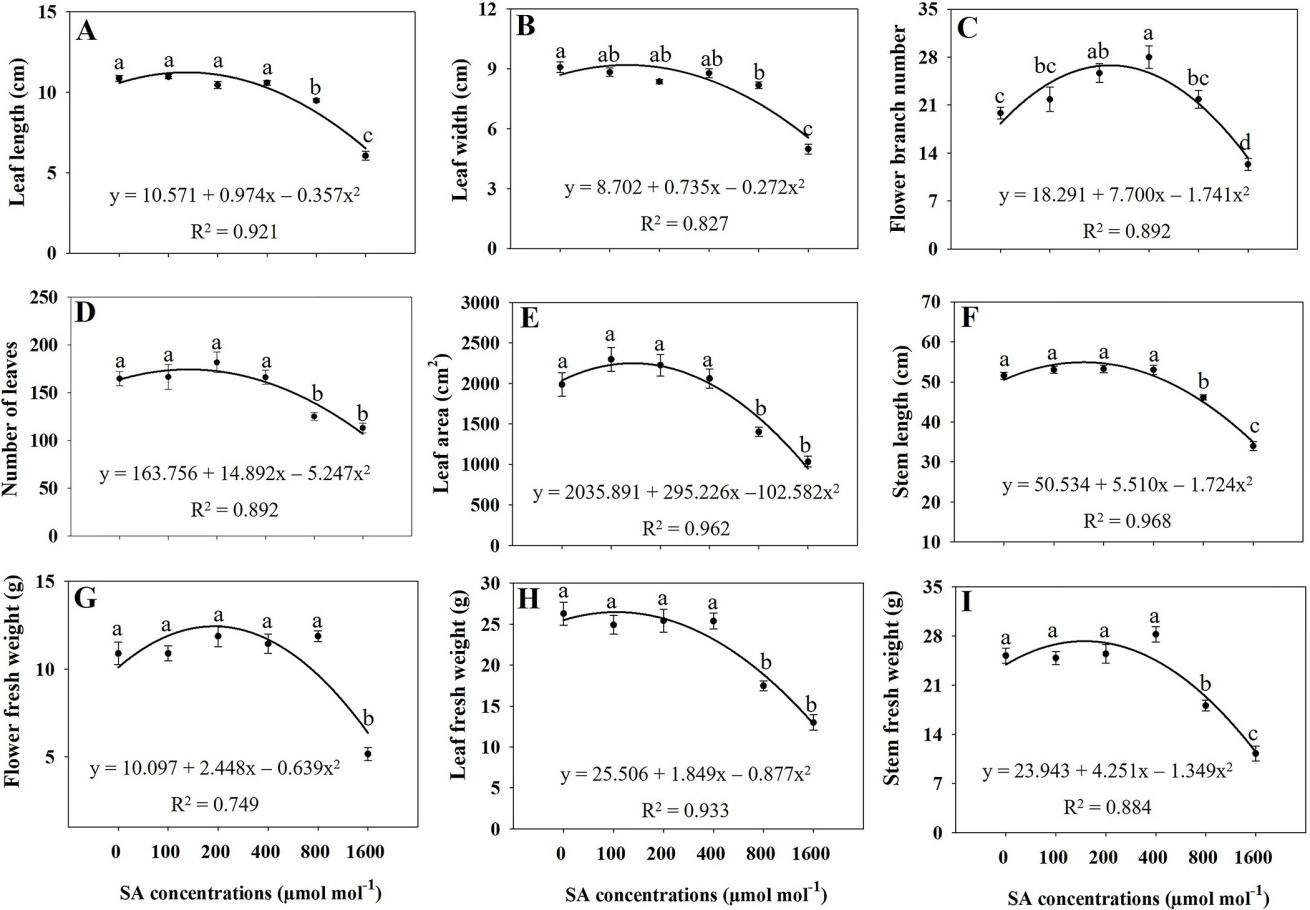
Regression equations for leaf length (A), leaf width (B), flower branch number (C), number of leaves (D), leaf area (E), stem length (F), flower fresh weight (G), leaf fresh weight (H), and stem fresh weight (I) of *A*. *rugosa* plants grown under different salicylic acid (SA) soaking treatments (0, 100, 200, 400, 800, and 1600 μmol mol^−1^ for 10 minutes). Each value represents the mean ± standard error (SE) of six samples (n = 6). Different letters represent the significant differences at *p* ≤ 0.05, as assessed using ANOVA, followed by Tukey’s multiple range test.

**Fig 3:**
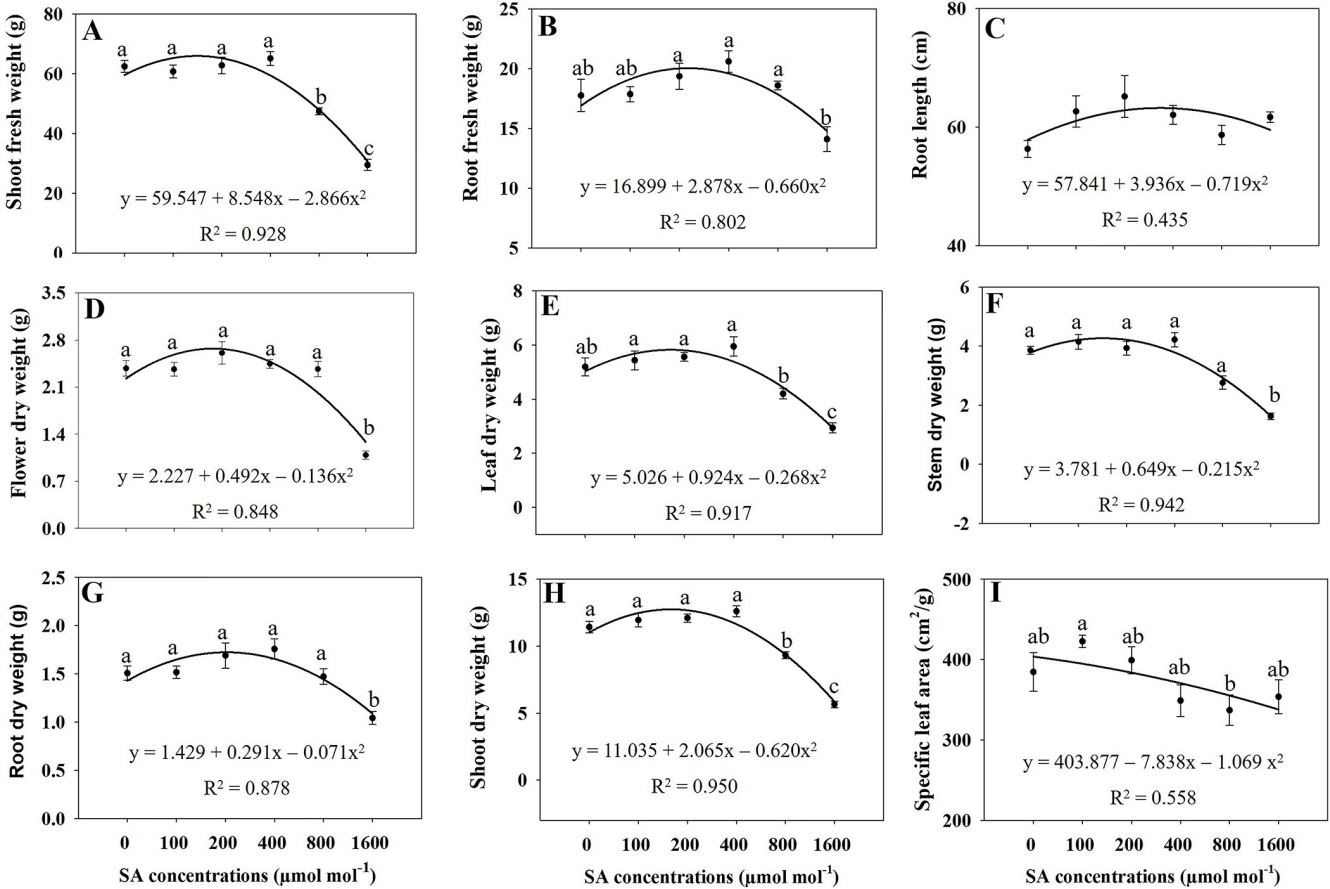
Regression equations for shoot fresh weight (A), root fresh weight (B), root length (C), flower dry weight (D), leaf dry weight (E), stem dry weight (F), root dry weight (G), shoot dry weight (H), and specific leaf area (I) of *A*. *rugosa* plants grown under different salicylic acid (SA) soaking treatments (0, 100, 200, 400, 800, and 1600 μmol mol^−1^ for 10 minutes). Each value represents the mean ± standard error (SE) of six samples (n = 6). Different letters represent the significant differences at *p* ≤ 0.05, as assessed using ANOVA, followed by Tukey’s multiple range test.

**Fig 4:**
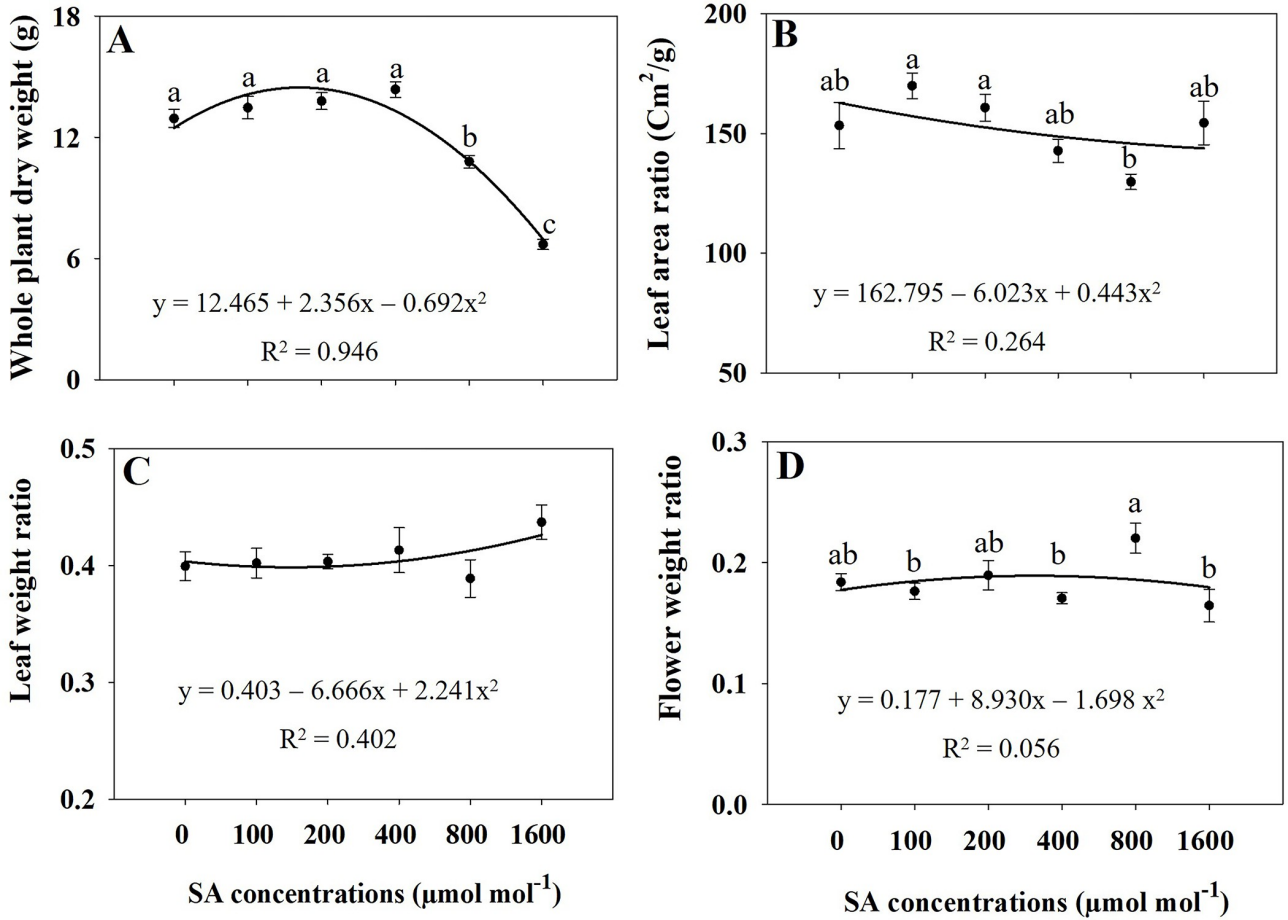
Regression equations for whole plant dry weight (A), leaf area ratio (B), leaf weight ratio (C), and flower weight ratio (D) of *A*. *rugosa* plants grown under different salicylic acid (SA) soaking treatments (0, 100, 200, 400, 800, and 1600 μmol mol^−1^ for 10 minutes). Each value represents the mean ± standard error (SE) of six samples (n = 6). Different letters represent the significant differences at *p* ≤ 0.05, as assessed using ANOVA, followed by Tukey’s multiple range test.

Regression analysis revealed that elevated concentrations of SA (specifically at 800 and 1600 μmol mol^−1^) induced significant oxidative stress, resulting in a substantial reduction in various plant growth variables (Figs 1–4). Under the influence of 800 and 1600 μmol mol^−1^ SA, all assessed growth variables, including leaf length, leaf width, leaf number, leaf area, stem length, leaf fresh weight, stem fresh weight, shoot fresh and dry weight, and whole plant dry weight exhibited a significant decrease (*p* ≤ 0.05) in comparison to the untreated treatment. Furthermore, administering 1600 μmol mol^−1^ of SA led to a significant reduction (*p* ≤ 0.05) on in the fresh weights of flowers, flower branch number, and the dry weights of flowers, leaves, stems, and roots in comparison to the control (Figs 1–4). The plant growth variables exhibited their lowest values when subjected to the 1600 μmol mol^−1^ SA treatment. Due to the application of 1600 μmol mol^−1^ SA, the shoot fresh weight experienced a substantial decrease of 52.86% compared to that in the untreated plants. The shoot dry weight and overall plant dry weight under the influence of 1600 μmol mol^−1^ SA were recorded at 50.48% and 48.22%, respectively, demonstrating a significant reduction (*p* ≤ 0.05) compared to that in the control plants. Under the 1600 μmol mol^−1^ SA, there was a notable decrease in the leaf area and stem length, with reductions of 42.16% and 34.11%, respectively, compared to that in the untreated treatment. The findings demonstrate that the application of 1600 μmol mol^−1^ SA had the most pronounced impact on diminishing the plant growth characteristics of *A*. *rugosa*.

### Chlorophyll, SPAD value, and total carotenoid content

**Fig 5:**
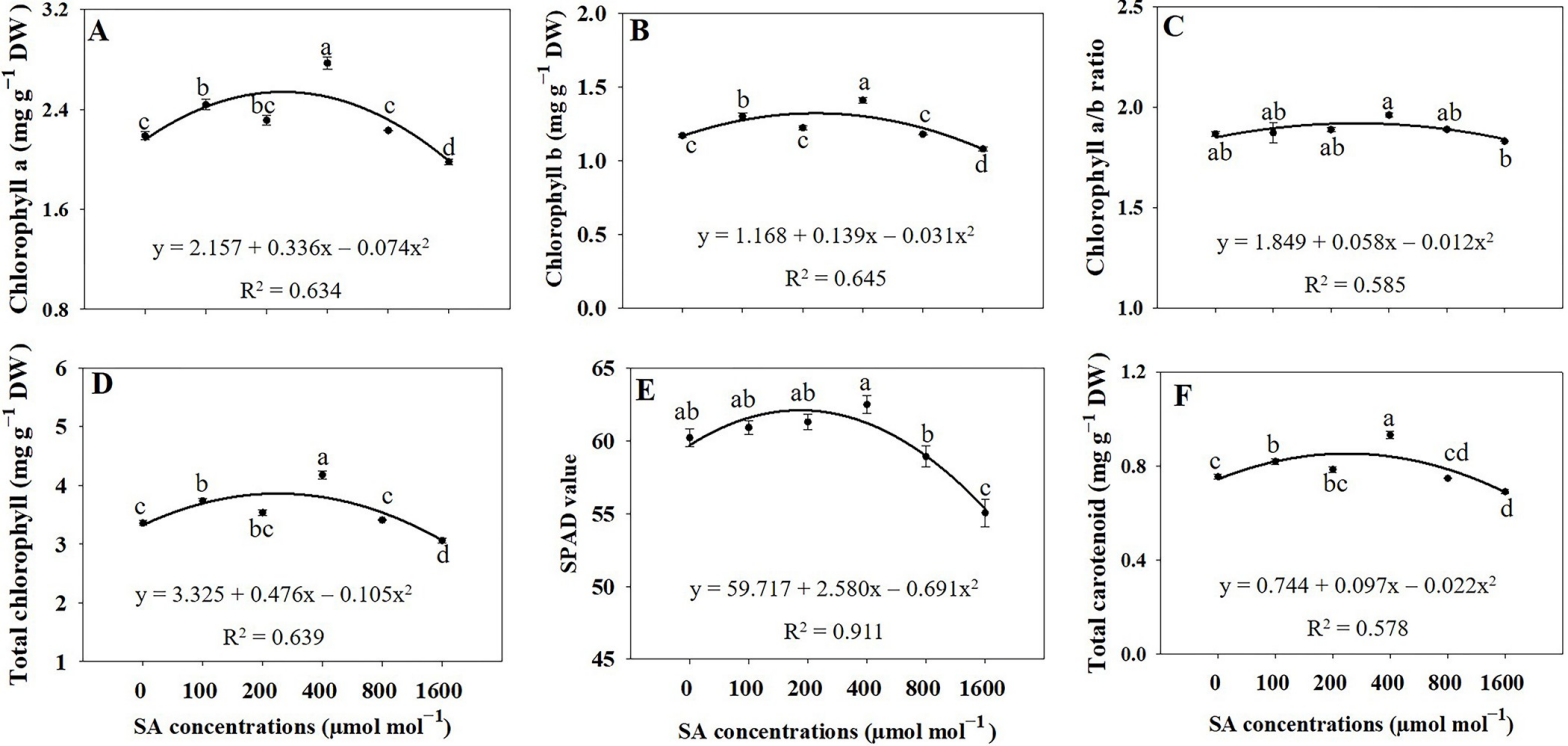
Regression equations for chlorophyll a levels (A), chlorophyll b levels (B), chlorophyll a/b ratio (C), total chlorophyll levels (D), soil plant analysis development (SPAD) value (E), and total carotenoid levels (F) in *A*. *rugosa* under different salicylic acid (SA) soaking treatments (0, 100, 200, 400, 800, and 1600 μmol mol^−1^ for 10 minutes). Each value represents the mean ± standard error (SE) of three samples (n = 3), except for the SPAD value, which is based on six samples (n = 6). Different letters represent the significant differences at *p* ≤ 0.05, as assessed using ANOVA, followed by Tukey’s multiple range test.

The regression analysis revealed significant increases (*p* ≤ 0.05) in Chl a and Chl b concentrations by 1.27-fold and 1.20-fold, respectively, under the 400 μmol mol^−1^ SA treatment compared to the untreated group (Fig 5A and 5B). Consequently, the 400 μmol mol^−1^ SA treatment exhibited the highest levels of total Chl and Chl l a/b ratio (Fig 5C and 5D). Additionally, the 400 μmol mol^−1^ SA treatment resulted in the highest SPAD value (Fig 5E). Regarding carotenoid content, treatments with 100 and 400 μmol mol^−1^ of SA showed significant increases (*p* ≤ 0.05) by 1.08-fold and 1.23-fold, respectively, compared to the control group (Fig 5F).

### Photosynthetic characteristics

**Fig 6:**
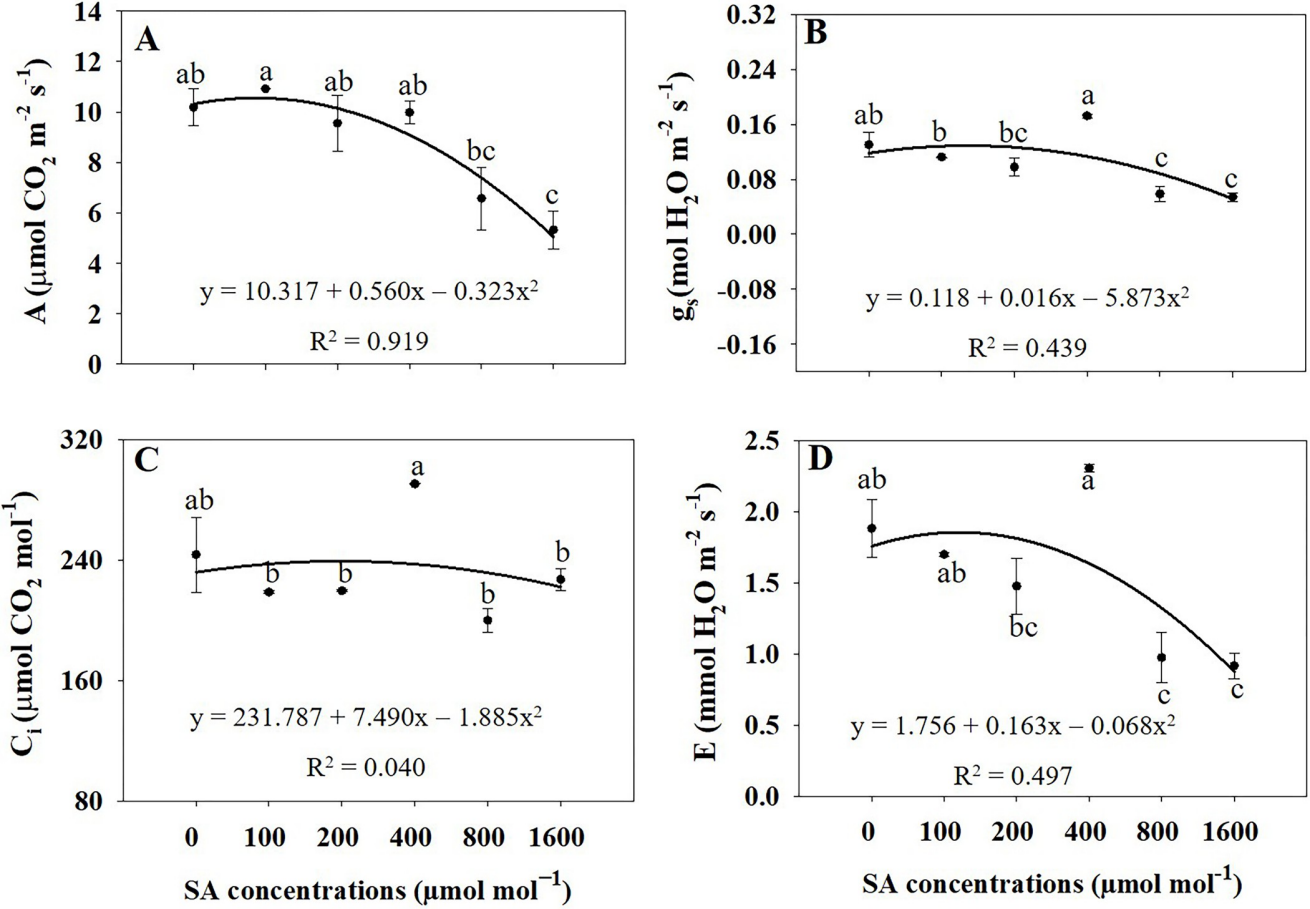
Regression equations for net photosynthetic rate (P_n_; A), stomatal conductance (g_s_; B), intercellular CO_2_ concentration (C_i_; C), and transpiration rate (E; D) of *A*. *rugosa* under different salicylic acid (SA) soaking treatments (0, 100, 200, 400, 800, and 1600 μmol mol^−1^ for 10 minutes). Each value indicates the mean ± standard error (SE) of three samples (n = 3). Different letters represent the significant differences at *p* ≤ 0.05, as assessed using the analysis of variance (ANOVA), followed by Tukey’s multiple range test.

Regression analysis revealed a distinct impact of SA treatment on the photosynthetic variables of *A*. *rugosa* (Fig 6). As SA concentrations increased to 800 and 1600 μmol mol^−1^, a decreasing trend in the net photosynthetic rate (A) was evident, with the lowest value observed under the 1600 μmol mol^−1^ treatment. Specifically, A at 1600 μmol mol^−1^ experienced a significant decrease (*p* ≤ 0.05) of 47.71% compared to untreated plants (Fig 6A). Similarly, stomatal conductance (gs) displayed a declining trend with higher SA concentrations, notably decreasing under treatments of 800 and 1600 μmol mol^−1^. These treatments led to significant decreases (*p* ≤ 0.05) of 54.91% and 58.31%, respectively, compared to the control group (Fig 6B). However, there was no notable disparity in intercellular CO_2_ concentration (C_i_) between the SA-treated groups and the control groups (Fig 6C). The transpiration rate (E) exhibited a significant decrease (*p* ≤ 0.05) under treatments of 800 and 1600 μmol mol^−1^ SA compared to the control group (Fig 6D).

### Total flavonoid, phenolic contents, and antioxidant capacity

**Fig 7:**
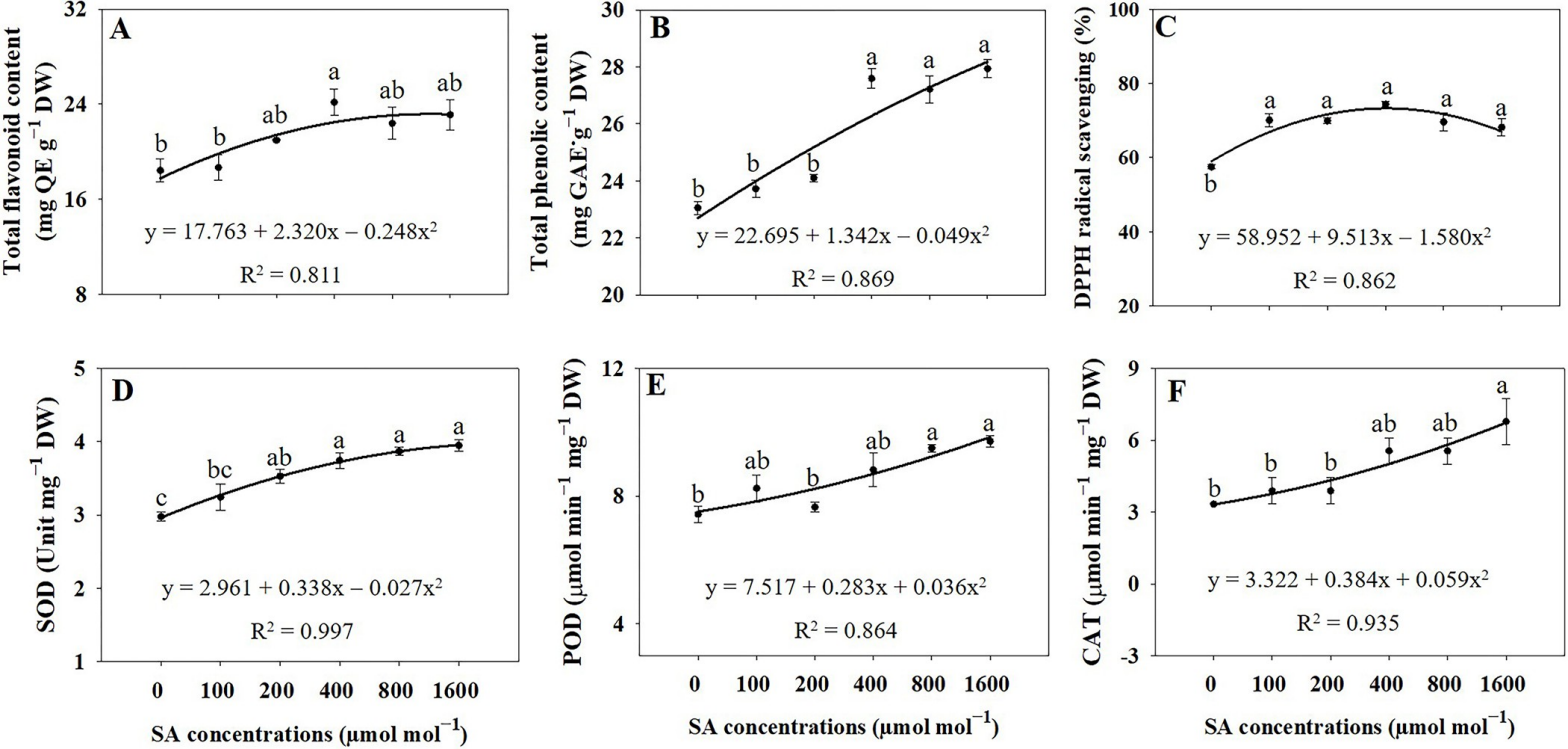
Regression equations for total flavonoid content (A), total phenolic content (B), 2,2-diphenyl-1-picrylhydrazyl (DPPH) radical scavenging activity (C), and superoxide dismutase (SOD) (D), peroxidase (POD) levels (E), and catalase (CAT) levels (F) of *A*. *rugosa* under different salicylic acid (SA) soaking treatments (0, 100, 200, 400, 800, and 1600 μmol mol^−1^ for 10 minutes). Each value indicates the mean ± SE of three samples (n = 3). Different letters represent the significant differences at *p* ≤ 0.05, as assessed using ANOVA, followed by Tukey’s multiple range test.

According to the regression analysis, the application of the 400 μmol mol^−1^ treatment resulted in a significant increase (*p* ≤ 0.05) in total flavonoid content, reaching levels 1.31 times higher than those of the control group, representing the highest observed values (Fig 7A). The total phenolic content exhibited a significant increase (*p* ≤ 0.05) in response to treatments of 400, 800, and 1600 μmol mol^−1^, showing elevations of 19.70%, 18.06%, and 21.20%, respectively, compared to the control group (Fig 7B). All treatments with SA significantly increased (*p* ≤ 0.05) DPPH radical-scavenging activity compared to that of the control plants (Fig 7C). The SOD enzyme activity showed a significant increase (*p* ≤ 0.05) when treated with SA at concentrations of 200, 400, 800, and 1600 μmol mol^−1^, resulting in elevations of 18.49%, 25.72%, 29.91%, and 32.61%, respectively, compared to that in the control group (Fig 7D). Moreover, the activity of the POD enzyme exhibited a significant increase (*p* ≤ 0.05), with enhancements of 27.82% and 30.69% observed under 800 μmol mol^−1^ and 1600 μmol mol^−1^, respectively, when compared to the control group (Fig 7E). Similarly, the CAT enzyme activity demonstrated a significant increase (*p* ≤ 0.05), reaching up to 2.03 times higher levels under 1600 μmol mol^−1^ compared to the control group (Fig 7F).

### Concentrations and contents of acacetin, tilianin, and RA

**Fig 8:**
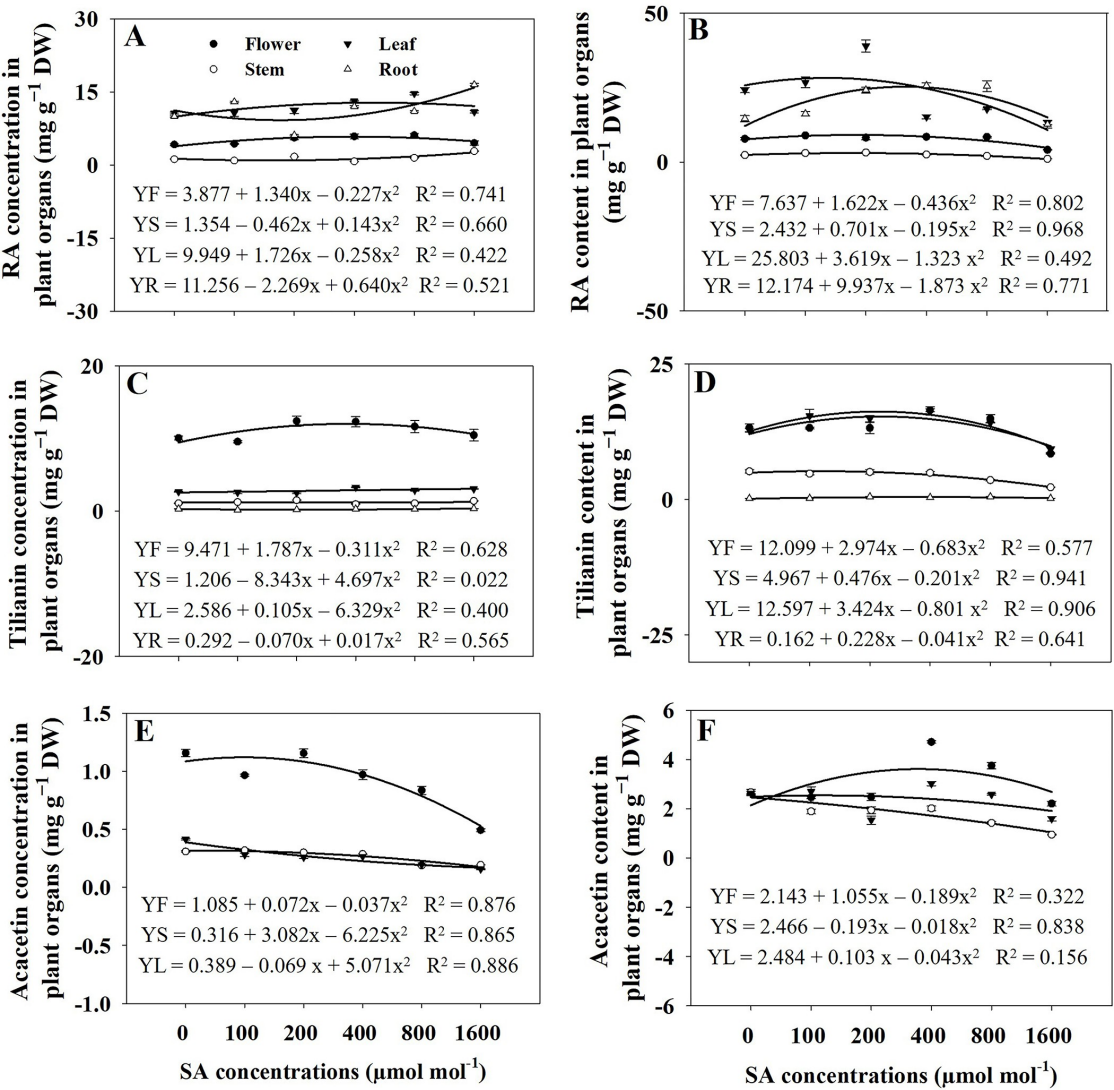
Regression equations for rosmarinic acid (RA) concentration (A) and content (B), tilianin concentration (C) and content (D), and acacetin concentration (E) and content (F) in plant organs of *A*. *rugosa* plants under different salicylic acid (SA) soaking treatments (0, 100, 200, 400, 800, and 1600 μmol mol^−1^ for 10 minutes). Each value indicates the mean ± SE of three samples (n = 3). F: flower, S: stem: L: leaf, R: root.

The concentrations of RA, tilianin, and acacetin varied among the different organs of *A*. *rugosa*. The roots had the highest concentration of RA, whereas the flowers had the highest concentrations of tilianin and acacetin. Acacetin concentration was found to be undetectable in the roots. The concentrations of RA in flowers and leaves were significantly increased (*p* ≤ 0.05) in response to the 400 and 800 μmol mol^−1^ SA treatments, respectively, compared to that in the control group. The highest concentration of RA was observed in the stems and roots under the 1600 μmol mol^−1^ SA treatment (Fig 8A). However, no significant difference in tilianin concentration in flowers was observed between the SA treatments and control groups. The stems exhibited the higher tilianin concentration under the 200 and 1600 μmol mol^−1^ SA treatment. In comparison, the leaves showed the highest tilianin concentration under the 400 μmol mol^−1^ SA treatment. Under 1600 μmol mol^−1^ SA treatment, the root exhibited the highest levels of tilianin concentration (Fig 8C). The elevated SA treatment demonstrated a tendency to reduce acacetin concentrations in the organs of *A*. *rugosa* plants compared to those in the control plants (Fig 8E).

Based on the regression analysis, variations in plant dry weights influenced the levels of RA content in flowers when exposed to an RA concentration of 1600 μmol mol^−1^ SA, resulting in the lowest levels observed. Stems exhibited the highest RA content under the treatment of 200 μmol mol^−1^, while leaves showed the highest RA content under the treatment of 400 μmol mol^−1^ (Fig 8B). Compared to the control group, roots showed higher RA and tilianin contents when subjected to treatments of 200, 400, and 800 μmol mol^−1^ SA, respectively (Fig 8B and 8D). Under an SA concentration of 400 μmol mol^−1^, the flowers and leaves exhibited the highest tilianin contents, respectively. The tilianin content in stems showed a significant decrease (*p* ≤ 0.05) under SA concentrations of 800 and 1600 μmol mol^−1^ compared to that in the control group. The leaves exhibited the lowest tilianin content when exposed to a concentration of 1600 μmol mol^−1^ SA. The content of tilianin in the roots exhibited a significant (*p* ≤ 0.05) rise when subjected to SA concentrations of 200, 400, and 800 μmol mol^−1^, in contrast to the control group (Fig 8D). The flowers exhibited the highest acacetin content when exposed to a concentration of 400 μmol mol^−1^ SA. Conversely, acacetin levels in the stems tended to decrease under SA treatment compared to those in the control group. The leaves exhibited the lowest acacetin content when exposed to a concentration of 1600 μmol mol^−1^ SA (Fig 8F).

**Fig 9:**
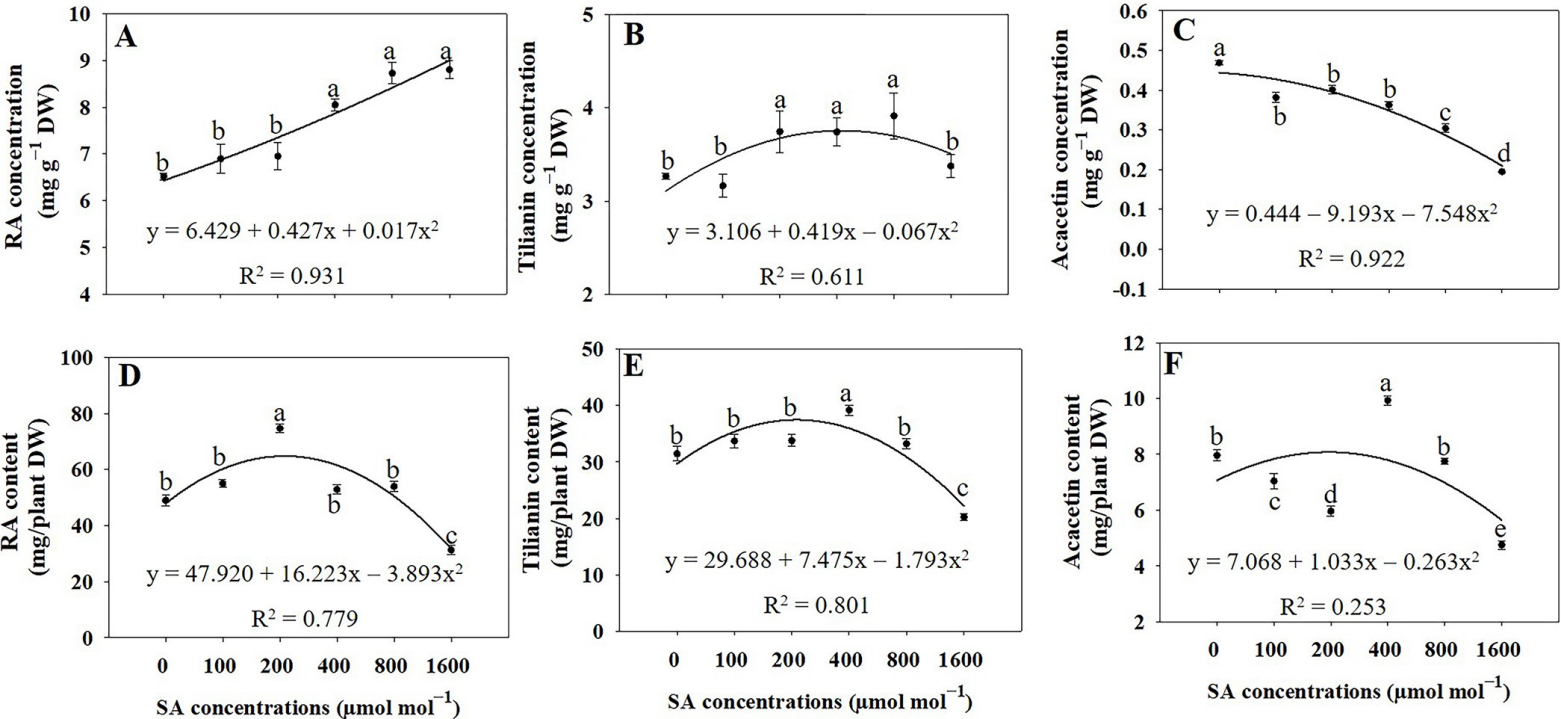
Regression equations for rosmarinic acid (RA) concentration (A) and content (D), tilianin concentration (B) and content (E), and acacetin concentration (C) and content (F) in *A*. *rugosa* whole plant under different salicylic acid (SA) soaking treatments (0, 100, 200, 400, 800, and 1600 μmol mol^−1^ for 10 minutes). Each value indicates the mean ± SE of three samples (n = 3). Different letters represent the significant differences at *p* ≤ 0.05, as assessed using ANOVA, followed by Tukey’s multiple range test.

Based on the regression analysis, the concentration of RA in entire plants subjected to SA treatments at concentrations of 400, 800, and 1600 μmol mol^−1^ significantly increased (*p* ≤ 0.05) by 23.59%, 33.86%, and 35.01%, respectively, compared to the untreated control group (Fig 9A). Conversely, the RA content in the entire plant reached its peak under 200 μmol mol^−1^ SA, exhibiting a remarkable increase of 52.46% higher than in the control group (Fig 9D). The plants treated with 200, 400, and 800 μmol mol^−1^ SA displayed a significant increase (*p* ≤ 0.05) in tilianin concentrations, and the increases were recorded as 14.62%, 14.55%, and 19.82% respectively, in comparison to that in the untreated control group (Fig 9B). Conversely, the SA application led to a significant reduction (*p* ≤ 0.05) in the concentration of acacetin in plants compared to that in the untreated control group (Fig 9C). Plants treated with 400 μmol mol^−1^ SA exhibited the highest values for tilianin and acacetin contents, with increases of 24.59% and 24.69%, respectively, compared to that in the untreated control group (Fig 9E and 9F).

### Concentrations and contents of acacetin 1, 2, and 3

**Fig 10:**
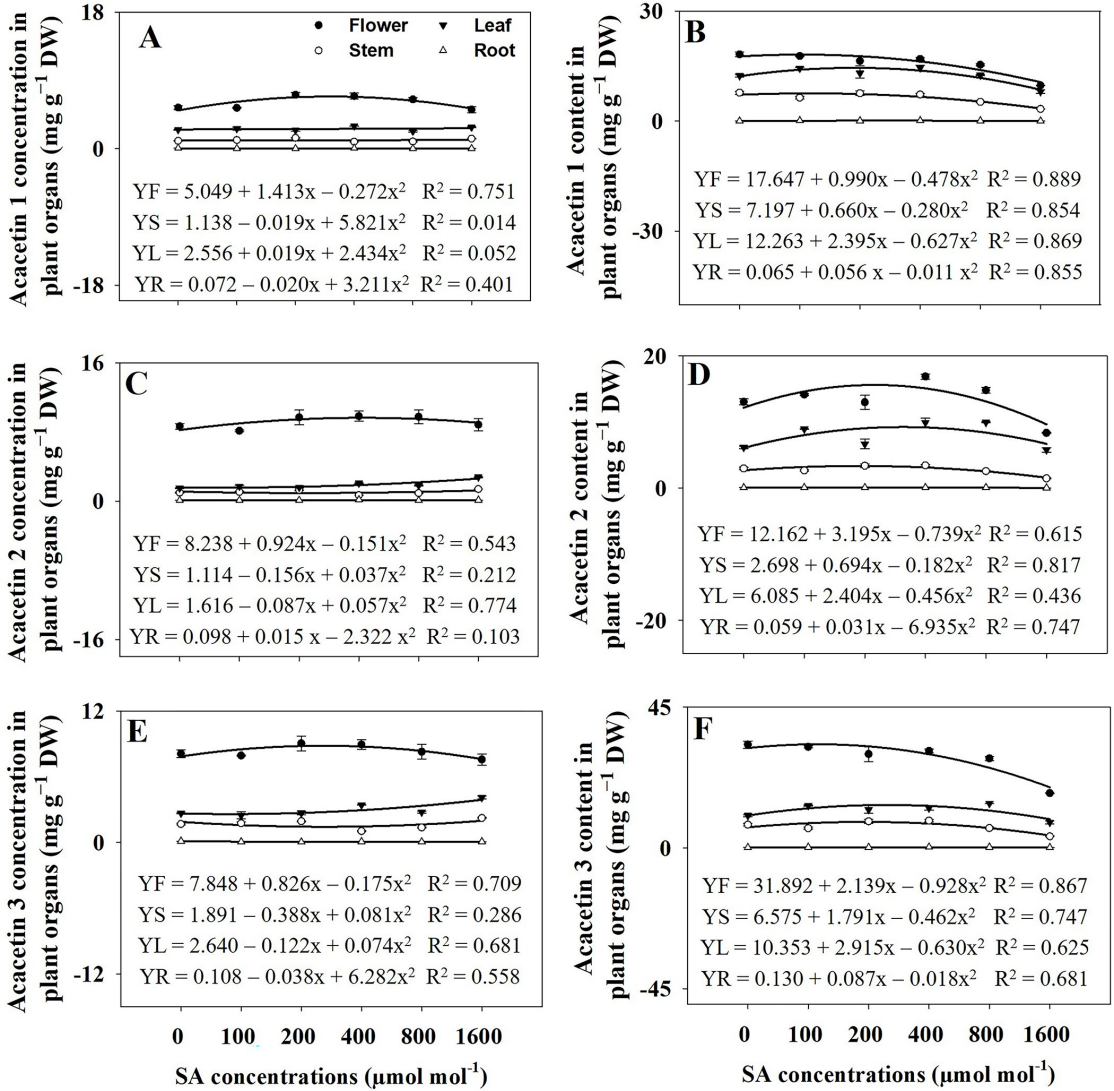
Regression equations for acacetin 7-O-2"-O-acetyl)β-D-glucopyranoside (acacetin 1; A and B), acacetin 7-O-(6"-O-malonyl)β-D-glucopyranoside (acacetin 2; C and D), and acacetin 7-O-(2"-O-acetyl-6"-malonyl)β-D-glucopyranoside (acacetin 3; E and F) concentrations and contents in plant organs of *A*. *rugosa* whole plant under different salicylic acid (SA) soaking treatments (0, 100, 200, 400, 800, and 1600 μmol mol^−1^ for 10 minutes). Each value indicates the mean ± SE of three samples (n = 3). F: flower, S: stem: L: leaf, R: root.

Based on the regression analysis, under 200 and 400 μmol mol^−1^ SA treatments, the concentration of acacetin 1 in flowers significantly increased (*p* ≤ 0.05) compared to untreated plants (Fig 10A). Significant increases (*p* ≤ 0.05) in the concentrations of acacetin 1 and 2 were observed in the stems when treated with 200 and 1600 μmol mol^−1^ SA compared to that of the untreated plants, respectively (Fig 10A and 10C). However, the concentration of acacetin 1 in leaves did not show a significant difference between the SA and control groups. The application of SA resulted in a significant reduction (*p* ≤ 0.05) in acacetin 1 and 3 concentrations in the roots compared to that in the control group (Fig 10A and 10E). No significant difference was observed in the concentrations of acacetin 2 and 3 in flowers between the SA and control groups. Under 400 and 1600 μmol mol^−1^ SA treatments, the concentration of acacetin 2 in leaves exhibited a significant increase (*p* ≤ 0.05) compared to those in the control group. The acacetin 2 concentration in roots was significantly higher (*p* ≤ 0.05) under 400 μmol mol^−1^ SA than that of the control group (Fig 10C). Moreover, the concentrations of acacetin 3 in both stems and leaves reached their highest levels under 1600 μmol mol^−1^ SA treatment (Fig 10A, 10C and 10E). However, due to a reduction in organ dry weight, the lowest values of acacetin 1, 2, and 3 contents in all plant organs were observed under the 1600 μmol mol^−1^ SA treatment (Fig 10B, 10D and 10F).

**Fig 11:**
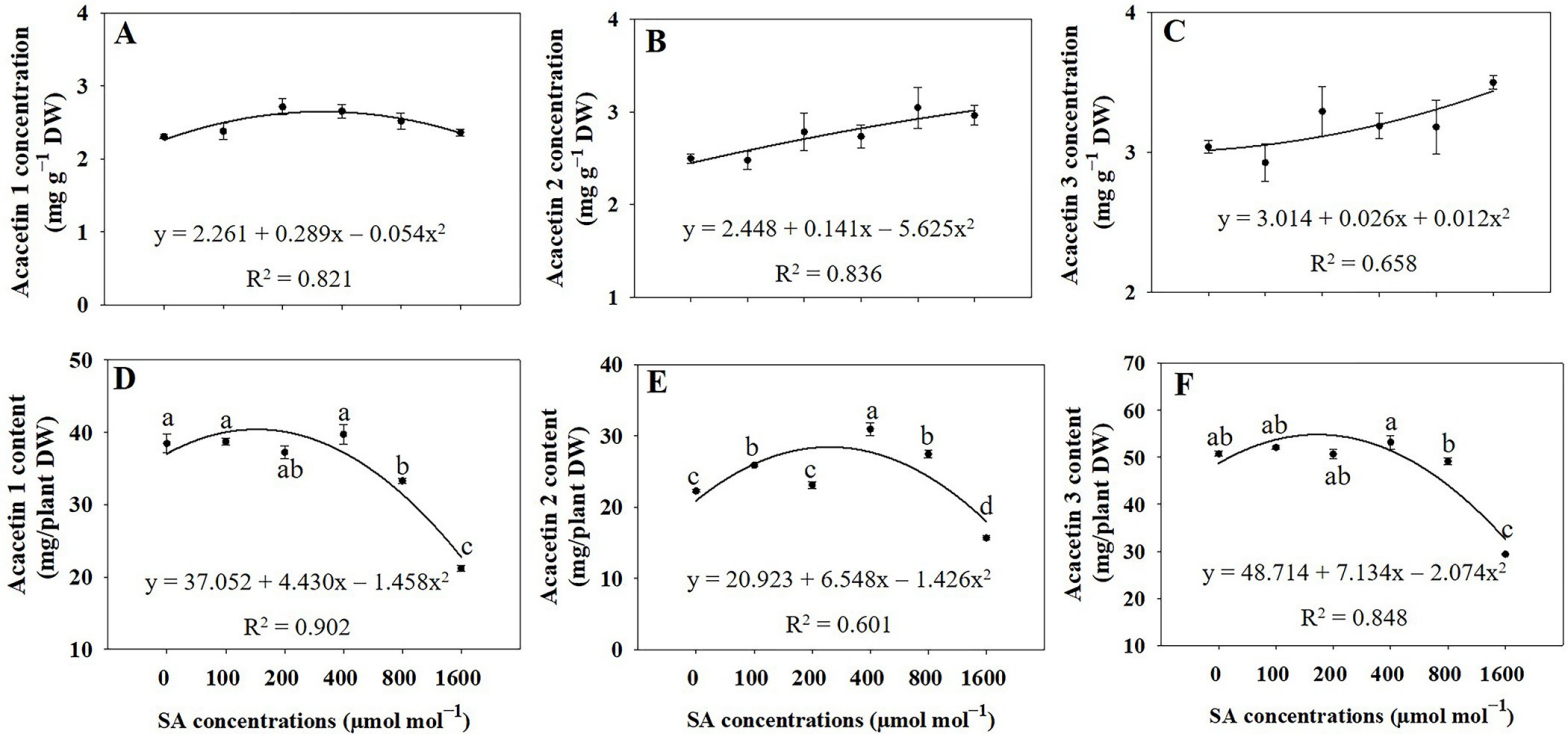
Regression equations for acacetin 7-O-2"-O-acetyl)β-D-glucopyranoside (acacetin 1; A and D), acacetin 7-O-(6"-O-malonyl)β-D-glucopyranoside (acacetin 2; B and E), and acacetin 7-O-(2"-O-acetyl-6"-malonyl)β-D-glucopyranoside (acacetin 3; C and F) concentrations and contents in *A*. *rugosa* whole plant under different salicylic acid (SA) soaking treatments (0, 100, 200, 400, 800, and 1600 μmol mol^−1^ for 10 minutes). Each value indicates the mean ± SE of three samples (n = 3). Different letters represent the significant differences at *p* ≤ 0.05, as assessed using ANOVA, followed by Tukey’s multiple range test.

No significant difference exists in acacetin 1, 2, and 3 concentrations in whole plants between the SA treatments and untreated plants (Fig 11A–11C). However, under the treatment of 400 μmol mol^−1^ SA, plants displayed the highest values for contents 1, 2, and 3, with increases of 1.03, 1.39, and 1.05 times, respectively, in comparison to that in the untreated control group (Fig 11D–11F).

## Discussion

### Plant growth variables

This study shows that elevated levels of SA at 800 and 1600 μmol mol^−1^ led to significant oxidative stress and a substantial reduction in plant growth. Various growth variables, including leaf length, leaf width, leaf number, leaf area, stem length, leaf fresh weight, stem fresh weight, shoot fresh and dry weight, and whole plant dry weight, exhibited a marked decrease under the influence of 800 and 1600 μmol mol^−1^ SA, compared to that in the untreated plants. Additionally, the administration of 1600 μmol mol^−1^ SA resulted in significant reductions in the fresh weights of flowers, the number of flower branches, and the dry weights of flowers, leaves, stems, and roots compared to those of the untreated plants. The plant growth variables were lowest when subjected to the 1600 μmol mol^−1^ SA treatment. These findings are consistent with previous studies that reported the negative impact of high SA levels on plant growth [29, 30]. Several studies have demonstrated that elevated SA concentrations can induce oxidative stress and disrupt various physiological and biochemical processes in plants. The oxidative stress caused by high SA levels can lead to reduced photosynthetic activity, impaired nutrient uptake, altered hormonal balance, and ultimately hindered plant growth and development [31, 32]. For example, the application of 50 μM SA significantly promoted the growth of chamomilla, whereas 250 μM SA led to a decrease in plant growth [33]. These results have important implications for the cultivation and management of *A*. *rugosa* plants. Therefore, it is crucial to carefully regulate SA levels to avoid excessive oxidative stress and promote optimal plant growth.

### Chlorophyll, SPAD value, and total carotenoid content

We observed a significant increase in Chl a and Chl b levels in the 100 and 400 μmol mol^−1^ SA treatment group compared to that in the group without SA treatment. Moreover, the 400 μmol mol^−1^ SA treatment resulted in the highest levels of total Chl, SPAD value, and Chl a/b ratio. These findings suggest that SA supplementation at this concentration positively influences the concentration of Chl and its ratio, potentially enhancing the photosynthetic efficiency of plants. Additionally, it enhances Chl synthesis in plants and can upregulate the expression of key enzymes involved in Chl biosynthesis, leading to an increase in Chl content [34, 35]. Furthermore, SA treatment improved the efficiency of Chl biosynthesis by enhancing the availability of precursor molecules and facilitating the transport of intermediates involved in Chl synthesis [35–37]. Applying 0.5 mM SA to mustard plants led to a noticeable increase in Chl fluorescence [35]. The levels of total Chl in Chinese chives were significantly increased with the application of SA at concentrations of 150 μM [38]. The previous study demonstrated that the application of 1 mmol SA for *Allium hirtifolium* significantly increased the levels of Chl a, Chl b, and total Chl compared to the control group [39]. Similarly, in this study, Chl a and b concentrations increased, contributing to higher total Chl levels in SA-treated plants. As with Chl, SA influences carotenoid synthesis in plants, and it stimulates the expression of genes encoding the enzymes involved in carotenoid biosynthesis, thereby promoting carotenoid accumulation [37]. SA treatment enhances the availability of precursors and cofactors required for carotenoid synthesis and increases the activity of key enzymes in the carotenoid biosynthetic pathway [34]. These processes led to an increase in the total carotenoid content in SA-treated plants. Likewise, the prior study revealed that applying 1 mmol of SA to *Allium hirtifolium* significantly enhanced the carotenoid levels in comparison to the control group [39].

### Photosynthetic characteristics

Compared to the control treatment, there was a decrease in the A under higher concentrations of SA treatments, particularly at 800 and 1600 μmol mol^−1^ SA. The observed decreasing trend in the net photosynthetic rate with higher SA concentrations suggests that elevated SA levels may have a negative impact on photosynthesis. Although SA is known for its roles in plant defense and stress responses, higher concentrations can interfere with photosynthetic processes or disrupt the functioning of the photosynthetic machinery [32]. This interference may lead to reduced CO_2_ fixation and a decrease in the net photosynthetic rate. The significant decrease of 47.71% in the net photosynthetic rate at 1600 μmol mol^−1^ SA compared to that in the untreated plants highlights the sensitivity of photosynthesis to extremely high SA concentrations. Such a reduction in the photosynthetic rate can have detrimental effects on plant growth and overall productivity, as it directly affects the plant’s energy production and ability to synthesize essential nutrients.

The observed decrease in g_s_ and E with higher SA concentrations (800 and 1600 μmol mol^−1^) suggests that elevated levels of SA might negatively impact stomatal behavior. SA is involved in plant stress responses and signaling pathways, including regulating stomatal apertures [40]. Higher SA concentrations may interfere with the hormonal signaling pathways that control stomatal opening and closure, reducing stomatal conductance. Such reductions in stomatal conductance may affect plant water-use efficiency, gas exchange, and overall physiological performance. The observed decrease in transpiration rate under the 800 and 1600 μmol mol^−1^ SA treatments suggests that higher concentrations of SA might lead to reduced stomatal conductance and subsequent decreased water loss through transpiration [40]. SA plays a role in the regulation of stomatal behavior. Higher SA concentrations may trigger stomatal closure, reducing the rate at which water vapor escapes from leaves [32]. This adaptive response to SA treatment could be an attempt by plants to conserve water and cope with potential stress conditions.

### Total flavonoid and phenolic contents, and antioxidant capacity

We observed that SA, specifically at a concentration of 400 μmol mol^−1^, has a potent elicitation effect on flavonoid biosynthesis in the *A*. *rugosa*. The significant increase in total flavonoid content in response to the 400 μmol mol^−1^ SA treatment suggests that SA acts as a signaling molecule, triggering the upregulation of genes and enzymes involved in flavonoid biosynthesis [41]. SA likely activates specific transcription factors or signaling pathways, leading to enhanced expression of flavonoid biosynthetic genes and, subsequently, higher flavonoid production [42]. Similarly, a previous study revealed that the concentration of flavonoids in *Agastache foeniculum* increased by 1.4 times when subjected to SA treatment compared to that in control plants [19]. The concentration-dependent response of SA to the total flavonoid content was notable. Among the tested SA concentrations, 400 μmol mol^−1^ was the most effective in increasing flavonoid levels. This concentration-dependent effect indicates that there may be an optimal range of SA concentrations for eliciting flavonoid biosynthesis, with concentrations lower or higher than the optimum being less effective.

However, Fig 4B showed that the total phenolic content in the plant exhibited a significant increase in response to specific concentrations of SA treatments, including 400, 800, and 1600 μmol mol^−1^, among the range of tested concentrations (0, 100, 200, 400, 800, and 1600 μmol mol^−1^ SA treatments). These findings align with earlier reports, where SA treatment led to a significant 2.5-fold increase in phenolic content in the *Ammi visnaga* L. plant compared to that in the control plants [43]. Furthermore, the total phenol content in table grapes exhibited a notable increase, rising from 768.3 mg^.^100 g^−1^ under control conditions to 1843.5 mg^.^100 g^−1^ when treated with 100 mg^.^ L^−1^ of SA [44]. The significant increase in total phenolic content in response to specific SA treatments suggests that SA acts as an elicitor, inducing the upregulation of genes and enzymes involved in the phenylpropanoid pathway and leading to increased phenolic compound synthesis [18]. SA, a widely recognized signaling molecule, plays a crucial role in plant defense responses and signaling pathways [13]. When applied externally, it can trigger plant defense mechanisms, producing secondary metabolites such as phenolics that aid in stress tolerance and protection.

Moreover, the results of this study demonstrated that all treatments with SA significantly increased DPPH radical scavenging activity compared to that in the control plants. Similarly, SA treatment augments DPPH radical scavenging activity in wheat [45]. This suggests that SA plays a crucial role in enhancing the antioxidant defense system of plants, potentially contributing to their ability to counteract oxidative stress. SA is a signaling molecule involved in various stress responses, including biotic and abiotic stressors [41]. Additionally, it has been reported to induce the expression of antioxidant enzymes and increase the production of non-enzymatic antioxidants, thereby contributing to ROS detoxification in plants [46]. The results of this study align with previous research that reported a positive effect of SA on the antioxidant defense systems of plants [47].

Superoxide dismutase (SOD) enzyme activity exhibited a significant increase at SA concentrations (200, 400, 800, and 1600 μmol mol^−1^) when compared to the control group. Peroxidase (POD) enzyme activity was significantly higher at SA concentrations of 800 μmol mol^−1^ and 1600 μmol mol^−1^ than the control group. Furthermore, catalase (CAT) enzyme activity showed a significant increase specifically at the highest SA concentration tested (1600 μmol mol^−1^) compared to the control group. The observed increase in SOD, POD, and CAT activities in response to specific SA treatments suggests that SA acts as an inducer, triggering the upregulation of genes and enzymes involved in SOD, POD, and CAT biosynthesis or activation [48]. The involvement of SA in the signaling pathways that govern antioxidant defense mechanisms was well established [37]. Several studies indicated the applications of SA played a role to increase SOD, POD, and CAT enzymes in some plant species such as *Salvia miltiorrhiza* [49], wheat [50], and cucumber [51]. Likewise, the previous study found that the application of 0.5, 0.75, and 1 mmol of SA to *Allium hirtifolium* resulted in significant enhancements in SOD and POD compared to the control group [39]. The highest level of SOD in *Cucurbita pepo* L was observed with the treatment of 1.5 mg/L SA [52]. When applied to plants, SA can activate specific defense responses, including the synthesis or activation of antioxidant enzymes such as SOD, POD, and CAT, to combat oxidative stress and protect the plant from damage caused by ROS. This highlights the role of SA as an effective inducer of antioxidant defense mechanisms, particularly SOD, POD, and CAT upregulation, which is critical in protecting plants from oxidative stress.

### Rosmarinic acid, tilianin, and acacetin concentrations and contents

The study investigated the impact of SA treatments on the concentrations of three important secondary metabolites, RA, tilianin, and acacetin in different organs of A. rugosa plants. The findings revealed organ-specific responses to SA treatment, highlighting the complexity of plant secondary metabolism regulation. Roots exhibited the highest concentration of RA, while tilianin and acacetin were most abundant in the flowers. Consistent with previous research [53], the study found that RA isomer was highest in roots compared with other organs of A. rugosa. Tilianin and acacetin were the highest amounts in flowers of A. rugosa [2]. This organ-specific distribution suggests distinct roles for these metabolites in different plant parts, possibly related to physiological functions or defense mechanisms. SA treatments led to significant increases in RA concentrations in flowers and leaves, with the highest concentration observed in stems and roots under the highest SA treatment (1600 μmol mol^−1^). This suggests a regulatory role of SA in promoting RA biosynthesis, particularly under stress conditions [54]. Unlike RA, tilianin concentrations did not show significant differences in flowers across SA treatments. Stems exhibited higher tilianin concentration under lower (200 μmol mol^−1^) and higher (1600 μmol mol^−1^) SA treatments, while leaves showed the highest concentration under moderate (400 μmol mol^−1^) SA treatment. This indicates a complex response of tilianin biosynthesis to SA treatment, possibly influenced by different signaling pathways or metabolic processes [55]. Regarding acacetin concentrations, under 200 and 400 μmol mol^−1^ SA treatments, the concentration of acacetin 1 in flowers significantly increased compared to untreated plants. Additionally, significant increases in the concentrations of acacetin 1 and 2 were observed in stems when treated with 200 and 1600 μmol mol^−1^ SA, respectively. However, the concentration of acacetin 1 in leaves was not significantly different between SA-treated and control groups, suggesting differential responses to SA across different plant tissues. SA application resulted in a significant reduction in acacetin 1 and 3 concentrations in roots compared to the control group, indicating a potential regulatory role of SA in acacetin biosynthesis or metabolism in root tissues. Notably, the concentrations of acacetin 2 and 3 exhibited varied responses to SA treatments across different plant organs, with significant increases observed under specific SA concentrations in leaves, roots, and stems. These findings highlight the intricate regulatory mechanisms underlying the accumulation of secondary metabolites, such as RA, tilianin, and acacetin, in A. rugosa, and provide insights into the potential role of SA in modulating their biosynthesis in a tissue-specific manner [18, 56].

The concentration of RA in the entire plant significantly increased when subjected to SA treatments at concentrations of 400, 800, and 1600 μmol mol^**−1**^, compared to that in the untreated control group (Fig 5A). The observed increase in RA content in response to specific SA treatments suggests that SA acts as an elicitor, triggering the upregulation of genes and enzymes involved in the phenylpropanoid pathway, leading to increased RA synthesis [18]. SA is a signaling molecule involved in plant defense responses and signaling pathways [13]. When applied externally, it can activate specific defense mechanisms in plants, producing secondary metabolites such as RA, which play essential roles in stress tolerance and protection. Fig 5B indicates that the plants treated with 200, 400, and 800 μmol mol^−1^ SA displayed a substantial increase in tilianin concentration compared to that in the untreated control group. The increased tilianin concentration in response to specific SA treatments suggests that SA acts as an inducer, triggering the upregulation of genes and enzymes involved in the flavonoid biosynthetic pathway and leading to increased tilianin synthesis [57]. When applied externally, it can activate specific defense mechanisms in plants, producing secondary metabolites such as tilianin, which contribute to stress tolerance and protection.

The results indicated that the application of SA treatments led to a significant reduction in the concentration of acacetin in plants compared with that in the untreated control group. The observed reduction in acacetin concentration in response to SA treatment suggests that SA may act as a suppressor or negative regulator of genes and enzymes involved in acacetin biosynthesis [57]. The involvement of SA in plant defense responses is well documented, and its application can induce the synthesis of diverse secondary metabolites such as phenolic compounds [14]. However, in the case of acacetin, SA treatment appeared to reduce biosynthesis or increase flavonoid degradation. The lack of a significant difference in acacetin concentrations between the SA treatments and untreated plants suggests that SA did not substantially impact the biosynthesis or accumulation of acacetin in plant tissues. This result implied that SA did not significantly influence the expression of genes or the activities of enzymes involved in specific biosynthetic pathways leading to acacetins 1, 2, and 3, and it is important to note that the absence of a significant difference in acacetin concentration does not necessarily indicate that SA does not affect the overall secondary metabolite profile of the plant. However, SA may influence the concentrations of other secondary metabolites, such as RA and tilianin, which play crucial roles in plant defense and stress responses. Nevertheless, depending on the overall dry weight of *A*. *rugosa* plants, the maximum RA content was observed under 200 μmol mol^−1^. The highest levels of tilianin, acacetin, and acacetin 1, 2, and 3 were recorded under 400 μmol mol^−1^ (Figs 9 and 11). Similarly, a prior study has reported that the application of SA resulted in elevated levels of hydroxycinnamic acids in six different species, concentrations of flavonoids in two species, total phenolic compound content in seven species, enhanced antioxidant activity in five species, as well as elevated levels of α-amylase in four species and increased α-glucosidase activity in five species within the Lamiaceae family when compared to control plants [19]. In *Ajuga integrifolia*, the highest enhancements in RA and caffeic acid content were observed at concentrations of 75 μM and 300 μM SA, respectively [58]. In the case of SA-treated *Fagonia indica*, there was a notable increase in the accumulation of phenolic content (12.29 μgGAE/mg) and flavonoid content (1.73 μgQE/mg) when compared to the control group [59]. The findings of this study provide a strong strategy for enhanced production of bioactive compounds in *A*. *rugosa*.

## Conclusion

This study offers valuable insights into the intricate interactions between SA and the developmental processes of *A*. *rugosa*. We showed that soaking the roots in SA concentrations of 200 and 400 μmol mol^−1^ resulted in increased contents of Chl, antioxidant enzymes, RA, acacetin, and tilianin without adversely affecting plant growth. The concentrations of RA, acacetin, and tilianin were determined in various plant organs (roots, flowers, stems, and leaves) to provide crucial information for future investigations into the quality of each organ. Nonetheless, further research is needed to comprehensively understand the underlying molecular mechanisms and signaling pathways involved in SA-mediated responses in *A*. *rugosa*. The findings of this study have promising implications for the practical application of SA in agriculture and horticulture, specifically in regulating plant growth and enhancing the production of valuable secondary plant metabolites that exhibit the potential to be applicable in a pharmaceutical context.
